# Resolution of synovitis and arrest of catabolic and anabolic bone changes in patients with psoriatic arthritis by IL-17A blockade with secukinumab: results from the prospective PSARTROS study

**DOI:** 10.1186/s13075-018-1653-5

**Published:** 2018-07-27

**Authors:** Eleni Kampylafka, Isabelle d’Oliveira, Christina Linz, Veronika Lerchen, Fabian Stemmler, David Simon, Matthias Englbrecht, Michael Sticherling, Jürgen Rech, Arnd Kleyer, Georg Schett, Axel J. Hueber

**Affiliations:** 1 Department of Internal Medicine 3 – Rheumatology and Immunology, Friedrich-Alexander-Universität Erlangen-Nürnberg (FAU) and Universitätsklinikum Erlangen, Ulmenweg 18, 91054 Erlangen, Germany; 2 Department of Dermatology, Friedrich-Alexander-Universität Erlangen-Nürnberg (FAU) and Universitätsklinikum Erlangen, Ulmenweg 18, 91054 Erlangen, Germany

**Keywords:** Psoriatic arthritis, Bone, Erosions, Enthesiophytes, Synovitis, bDMARDs

## Abstract

**Background:**

Although the effects of interleukin-17A (IL-17A) inhibition on the signs and symptoms of psoriatic arthritis (PsA) are well defined, little is known about its impact of local inflammatory and structural changes in the joints. The PSARTROS study was designed to elucidate the effects of IL-17A inhibition on inflammation and bone changes in joints affected by PsA.

**Methods:**

This was a prospective open-label study in 20 patients with active PsA receiving 24 weeks of treatment with the IL-17A inhibitor secukinumab. Magnetic resonance imaging (MRI), power Doppler ultrasound (PDUS), and high-resolution peripheral quantitative computer tomography (HR-pQCT) of the hands were performed at baseline and after 24 weeks to assess synovitis, periarticular inflammation, bone erosion, enthesiophyte formation, and bone structure. Demographic and clinical measures of joint disease (DAPSA and DAS28-ESR), skin disease (PASI and BSA), and composite measures (minimal disease activity, or MDA) were also recorded.

**Results:**

Treatment with secukinumab led to significant improvement of signs and symptoms of PsA; 46% reached MDA and 52% DAPSA low disease activity. MRI synovitis (*P* = 0.034) and signal in PDUS (*P* = 0.030) significantly decreased after 24 weeks of treatment. Bone erosions in MRI and HR-pQCT and enthesiophytes in the HR-pQCT did not show any progression, and structural integrity and functional bone strength remained stable.

**Conclusions:**

IL-17 inhibition by secukinumab over 24 weeks led to a significant decrease of synovial inflammation and no progression of catabolic and anabolic bone changes in the joints of patients with PsA.

**Trial registration:**

ClinicalTrials.gov Identifier: NCT02483234, June 26, 2015; retrospectively registered.

**Electronic supplementary material:**

The online version of this article (10.1186/s13075-018-1653-5) contains supplementary material, which is available to authorized users.

## Background

Psoriatic arthritis (PsA) is a chronic inflammatory disease affecting the joints and the entheses and leads to bone damage [1]. In the joints, the inflammatory process in PsA afflicts the synovium and the periosteal insertions of tendons and ligaments. Chronic inflammation at these synovial and entheseal sites leads to bone erosions and enthesiophytes, respectively, and a link to the intestinal microbiome has been suggested [1, 2]. Cytokines are considered to trigger both the inflammatory and structural changes in patients with PsA and to provide the essential link between inflammation and damage [3, 4]. Aside from tumor necrosis factor (TNF), interleukin-17A (IL-17A) has been identified as a key cytokine in psoriatic skin and joint disease [5]. Inhibition of IL-17A by neutralizing antibodies has been shown to improve the signs and symptoms of joint disease in patients with PsA [6–8].

Despite unequivocal evidence that IL-17 inhibition works on the signs and symptoms of PsA, its local effects on the joint are inadequately characterized. Radiographic studies suggest that IL-17A inhibition retards the progression of bone erosion; however, the overall progression rate in conventional radiographs is low in PsA [9]. Although the reduction of the burden of synovitis seems to be crucial for achieving protection from bone erosion, it is not fully clear whether these targets are indeed achieved by IL-17A inhibition. On the other hand, no data are available on whether IL-17A inhibition retards or arrests the progression of enthesiophyte formation in PsA. In this context, reduction of enthesitis appears to be important [10].

Preclinical data have suggested that Il-17 is a cytokine that modulates bone structure during inflammation. Thus, IL-17 increases osteoclast differentiation and mediates bone erosion in experimental arthritis by upregulating receptor activator of nuclear factor-kappa Β ligand (RANKL) and IL-1 [11, 12]. Furthermore, IL-17–producing TH17 cells induce osteoclast differentiation [13, 14]. These observations provide a rationale for the protection from bone erosion by IL-17 inhibition in PsA. Additional data suggest that the IL-23–IL-17 pathway is involved in enthesial inflammation, which is linked to new bone formation in PsA and axial spondyloarthritis [10, 15–18]. Finally, IL-17 has been shown to be a key mediator for bone loss in psoriatic disease both in mice and in humans [19]. PsA has been shown to be associated with increased fracture risk, which is likely triggered by the prolonged effect of inflammatory cytokines such as IL-17 [20]. Hence, the ideal protection from structural bone damage in PsA would mean an arrest of bone erosion, retardation of enthesiophyte formation, and maintenance of bone architecture and strength.

In this work, we performed a meticulous assessment of joints and bones of patients with PsA treated with secukinumab, a fully human monoclonal antibody selective for IL-17A, for 24 weeks. Patients with active PsA were enrolled in the prospective observational PSARTROS imaging study. Assessments were carried out by a combination of magnetic resonance imaging (MRI), power Doppler ultrasound (PDUS), and high-resolution peripheral quantitative computed tomography (HR-pQCT) to assess synovitis, periarticular inflammation, bone erosions, enthesiophytes, and bone architecture and strength at baseline and 24 weeks of secukinumab treatment. In addition, the clinical effects of secukinumab on the signs and symptoms of PsA were recorded.

## Methods

### Study design and patients

The PSARTROS study (ClinicalTrials.gov Identifier: NCT02483234) is a single-arm prospective exploratory open-label study to assess the effects of secukinumab treatment on the inflammatory and structural changes in the joints of patients with PsA. All patients received subcutaneous treatment with 300 mg secukinumab once weekly for the first five applications and then once monthly for a total of 24 weeks. Eligible patients were above 18 years of age and had been diagnosed with PsA according to the Classification Criteria for Psoriatic Arthritis (CASPAR) for at least 6 months prior to their inclusion in the study and had active joint disease with at least three tender and three swollen joints out of a 78/76-joint count. Corticosteroid treatment was allowed at stable doses of not more than 10 mg/day prednisone for at least 2 weeks before baseline and had to remain on a stable dose until the end of the study. Concomitant non-biologic treatment was allowed if on a stable dose for at least 4 weeks before randomization and throughout the study. Previous TNF inhibitor therapy was allowed after appropriate washout periods. Patients who had previously used drugs targeting IL-17 or IL-23p40, had received any recent live vaccines, had a history of tuberculosis, were pregnant, were using opioid analgesics, had a history of alcohol or drug abuse within the last 6 months, or had any uncontrolled medical condition were not eligible. All patients provided written informed consent, and institutional review boards/ethics committees approved the protocol.

### Magnetic resonance imaging

MRI was used to assess inflammatory and structural changes in the joints. MRI scans of the dominant hand were performed at baseline and after 24 weeks of secukinumab treatment by using a 1.5 T Magneton Avanto system (Siemens, Erlangen, Germany) as described before [21]. T1-weighted images with and without contrast agent as well as T2-weighted coronal fat saturated turbo inversion recovery magnitude (TIRM) sequences were assessed for synovitis, periarticular inflammation, tenosynovitis, bone erosions, and bone proliferations as well as osteitis. T1-weighted coronal images without contrast were used to assess erosions and bone proliferation. T1-weighted axial images after intravenous gadolinium injection were used to assess synovitis and tenosynovitis. The same T1-weighted images were assessed to evaluate periarticular inflammation. TIRM sequences were used to assess osteitis. Images were evaluated by two independent assessors (IO and CL) blinded to the identity of the patients and the sequence of the images using standardized Psoriatic Arthritis Magnetic Resonance Imaging Score–OMERACT (PsAMRIS-OMERACT) scoring [22].

### Power Doppler ultrasound

PDUS was used to assess the inflammatory changes in the joints. PDUS was performed at baseline and after 24 weeks of secukinumab treatment by using a Mylab twice ultrasound machine (Esaote Biomedica, Genova, Italy) with a 6- to 18-MHz probe (LA435). In 28 joints, comprising the metacarpophalangeal (MCP) 2–5, proximal interphalangeal (PIP) 2–5, wrist, knee and metatarsophalangeal (MTP) 2–5 joints of both sides, synovial hypertrophy and power Doppler signals were scored in accordance with the Global OMERACT EULAR Ultrasound Synovitis Score (GLOESS) [23, 24]. In this score, synovial hypertrophy and power Doppler signals are graded as absent (0), mild (1), moderate (2), or severe (3). In addition, the presence of effusion was assessed. Scans were performed and evaluated by an experienced sonographer (AK).

### High-resolution peripheral quantitative computed tomography

HR-pQCT was used to assess the structural changes in the joints and the bone composition and function. HR-pQCT of the dominant hand was performed at baseline and after 24 weeks of secukinumab treatment by using an XtremeCT I scanner (Scanco Medical, Brüttisellen, Switzerland). Scans were performed at MCP joints 2 and 3, PIP joints 2 and 3, and the distal radius. MCPs and PIPs were evaluated for erosions (numbers and volume) by using HR-pQCT Software [25] and enthesiophytes in accordance with the following grading: grade 1: maximum height ≤ 4 mm; grade 2: maximum height > 4 mm; and grade 3: diffuse osteoproliferation [26]. The MCP 2 and MCP 3 joints were additionally evaluated for the volume of erosions present by using the HR-pQCT Software [25]. The distal radius was evaluated for bone structural and microstructural parameters as described previously [20]. In addition, biomechanical properties of bone (stiffness and failure load) were assessed through micro-finite element analysis of the radius (FAIM software, version 8.0, Numerics88 Solutions Ltd., Calgary, AB, Canada) [27].

### Clinical assessments

Clinical evaluation at baseline and 24 weeks included tender joint counts (TJCs) 78, swollen joint counts (SJCs) 76, and visual analogue scales for patients and physicians global disease activity and patients pain. Activity of skin disease was assessed by psoriasis area severity index (PASI) and body surface area (BSA). Activity of PsA and response in signs and symptoms of PsA was assessed by Disease Activity in PSA (DAPSA) [28], Disease Activity Score (DAS) 28, Minimal Disease Activity (MDA) [29], and Psoriatic Arthritis Response Criteria (PsARC) as well as by American Colleague of Rheumatology (ACR) 20, 50, and 70 responses.

### Statistical analysis

The hypothesis of the study was that secukinumab treatment (a) leads to significant improvement of synovitis and periarticular inflammation and (b) arrests progression of both erosions and enthesiophytes in the joints of PsA patients after 24 weeks. The Wilcoxon signed-rank test was used for paired comparisons between baseline and week 24. Cross-sectional analyses were performed by using the Mann–Whitney *U* test for differences and Spearman correlation for relations. Statistical significance was set at *P* ≤ 0.05, and data were presented as median and quartiles. All analyses were performed in a two-tailed manner by using IBM SPSS version 21 (IBM, Armonk, NY, USA).

## Results

### Patient characteristics

In total, 20 patients with PsA were prospectively included in the study, and 60% of them were females. The median age was 50.5 years (interquartile range (IQR) 44–59), and median disease duration was 5.5 (1.25–11.75) years. Patients had moderate-to-high PsA activity according to Disease Activity Score 28-joint count–erythrocyte sedimentation rate (DAS28-ESR) (median 4.94; IQR 4.31–5.8) and DAPSA (median 27.55; IQR 22.53–38.35) with a median TJC of 10 (6.25–20) and SJC of 4 (3–5.75). Both oligo- and poly-articular PsA patients were included, and all patients showed clinical and imaging involvement of the hands (Additional file 1: Table S1). Skin involvement was low; PASI was 0.4 (0.2–1.9) and BSA was 0.4 (0.2–1.5); 55% of the patients were biological disease-modifying antirheumatic drug (bDMARD)–naïve entering secukinumab treatment, while 45% had previously experienced TNF inhibitor. Forty percent had concomitant conventional DMARD (cDMARD) treatment, which was kept stable during the treatment phase. Three patients dropped out during the 24-week study period: one due to recurrent pharyngitis, one due to lack of efficacy, and one due to consent withdrawal. Treatment with secukinumab showed an overall good safety profile, and no serious adverse events, including infection requiring hospitalization, were reported; neither deaths nor major lab abnormalities or injection site reactions occurred. The longitudinal imaging evaluation was performed in the 17 patients completing the study.

### Baseline imaging features and comparison of imaging modalities

**Fig. 1:**
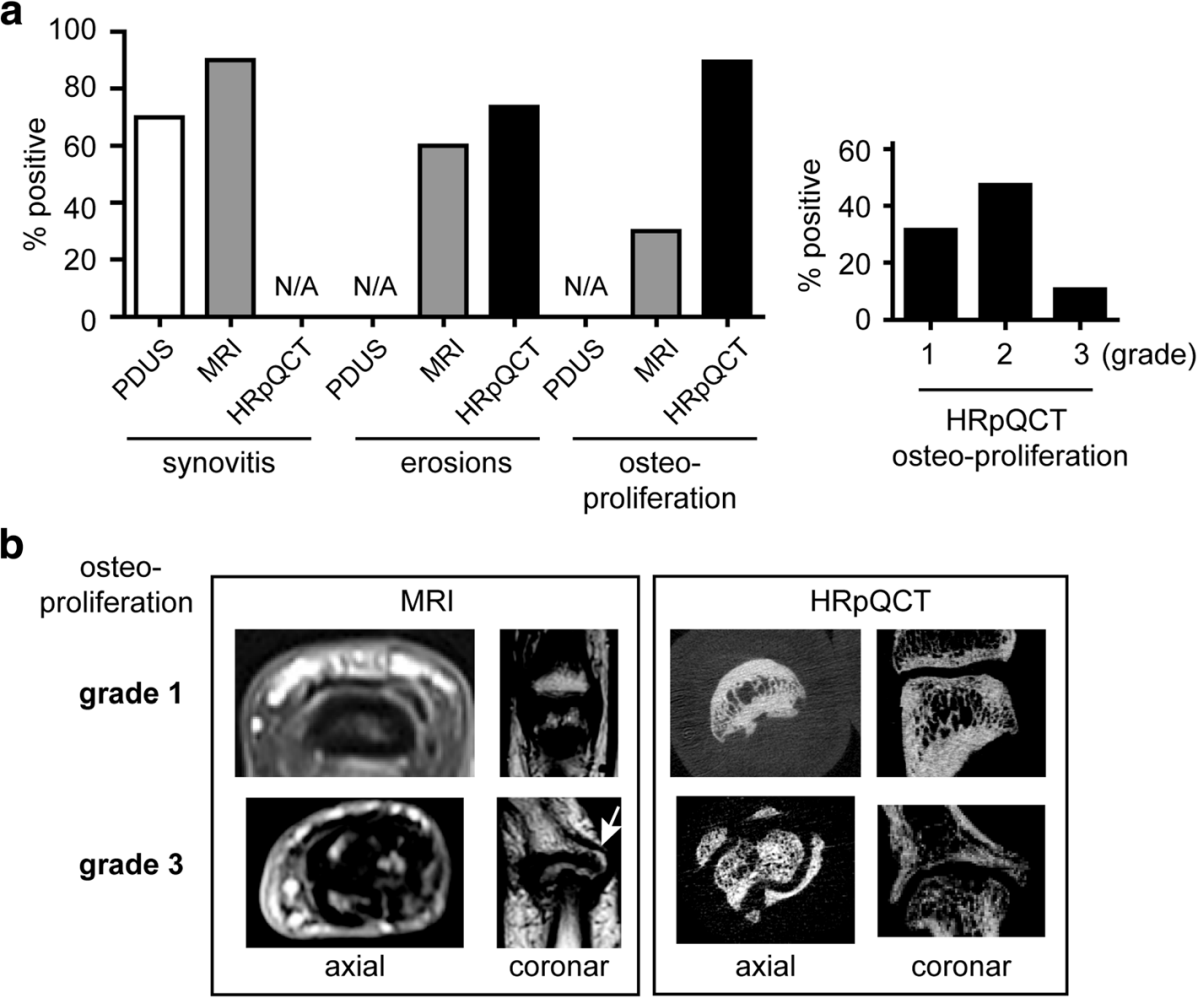
Prevalence of inflammatory and structural changes in the joints of patients with psoriatic arthritis. **a** On left, baseline imaging analysis and comparative presentation of the prevalence of synovitis, bone erosions, and enthesiophytes in power Doppler ultrasound (PDUS), magnetic resonance imaging (MRI), and high-resolution peripheral quantitative computed tomography (HR-pQCT); on right, prevalence of enthesiophytes according to size using HR-pQCT (grade 1: height < 4 mm, grade 2: height ≥ 4 mm, grade 3: diffuse proliferation). **b** Coronal and axial images of the same enthesiophytes (upper panel: grade 1, lower panel: grade 3) in MRI and HR-pQCT. Arrow indicates the lesion. Abbreviation: *N/A* not applicable

Baseline MRI investigation revealed synovitis in the vast majority (90%) of patients. Synovitis was also found in the majority of patients (70%) when using PDUS (Fig. 1a, Table 1). The median total PSAMRIS score was 5.5 (IQR 3–19.5), the median global OMERACT score was 8.5 (IQR 4.25–12.75), and MRI showed other forms of inflammation such as tenosynovitis, periarticular inflammation, and osteitis in a substantial number of patients (Table 1). The majority of patients with PsA had erosions in the MRI at baseline (60%) and this number was even higher when assessing the joints by HR-pQCT (73.7%). Enthesiophytes were found in only 30% of the patients when MRI was used, but these bone lesions were much more detectable by HR-pQCT, where most patients with PsA (89%) showed enthesiophytes. The majority of enthesiophytes were graded as mild (grade 1) or moderate (grade 2) and only few were severe (grade 3), which were then also detected by MRI (Fig. 1b).

**Table 1:** Baseline imaging characteristics

Magnetic resonance imaging	
Synovitis %	90%
PSAMRIS Synovitis (median, IQR)	2.5 (1.25, 6)
Osteitis %	20%
PSAMRIS Osteitis (median, IQR)	0 (0, 0)†
PSAMRIS Osteitis (mean ± SD)	1 ± 2.8
Erosion %	60%
PSAMRIS Erosion (median, IQR)	1 (0, 2.75)
Proliferation %	30%
PSAMRIS Proliferation (median, IQR)	0 (0, 1)†
PSAMRIS Proliferation (mean ± SD)†	1.1 ± 2.4
Periarticular %	25%
PSAMRIS Periarticular (median, IQR)	0 (0, 1.5)†
PSAMRIS Periarticular (mean ± SD)†	0.8 ± 1.6
Tenosynovitis %	35%
PSAMRIS Tenosynovitis (median, IQR)	0 (0, 1.75)†
PSAMRIS Tenosynovitis (mean ± SD)†	1.6 ± 2.9
Total PSAMRIS (median, IQR)	5.5 (3, 19.5)
PDUS	
Synovitis %	70%
OMERACT Hypertrophy (median, IQR)	5.5. (2.25, 10.75)
OMERACT Effusion (median, IQR)	4.5 (1, 8.5)
OMERACT Power Doppler (median, IQR)	2 (1.25, 5.75)
OMERACT Global (median, IQR)	5 (3, 11)
HR-pQCT	
Erosions (%)	73.7%
Enthesiophytes (%)	89.5%

*HR-pQCT* high-resolution peripheral quantitative computed tomography, *IQR* interquartile range, *OMERACT* outcome measures in rheumatoid arthritis clinical trials, *PDUS* power Doppler ultrasound, *PSAMRIS* psoriatic arthritis magnetic resonance imaging scoring system. †Additionally reported as mean and standard deviation (mean ± standard deviation) because median was equal to zero. Data are based on all 20 psoriatic arthritis patients recruited into the study

### Effects of secukinumab on joint inflammation

**Fig. 2:**
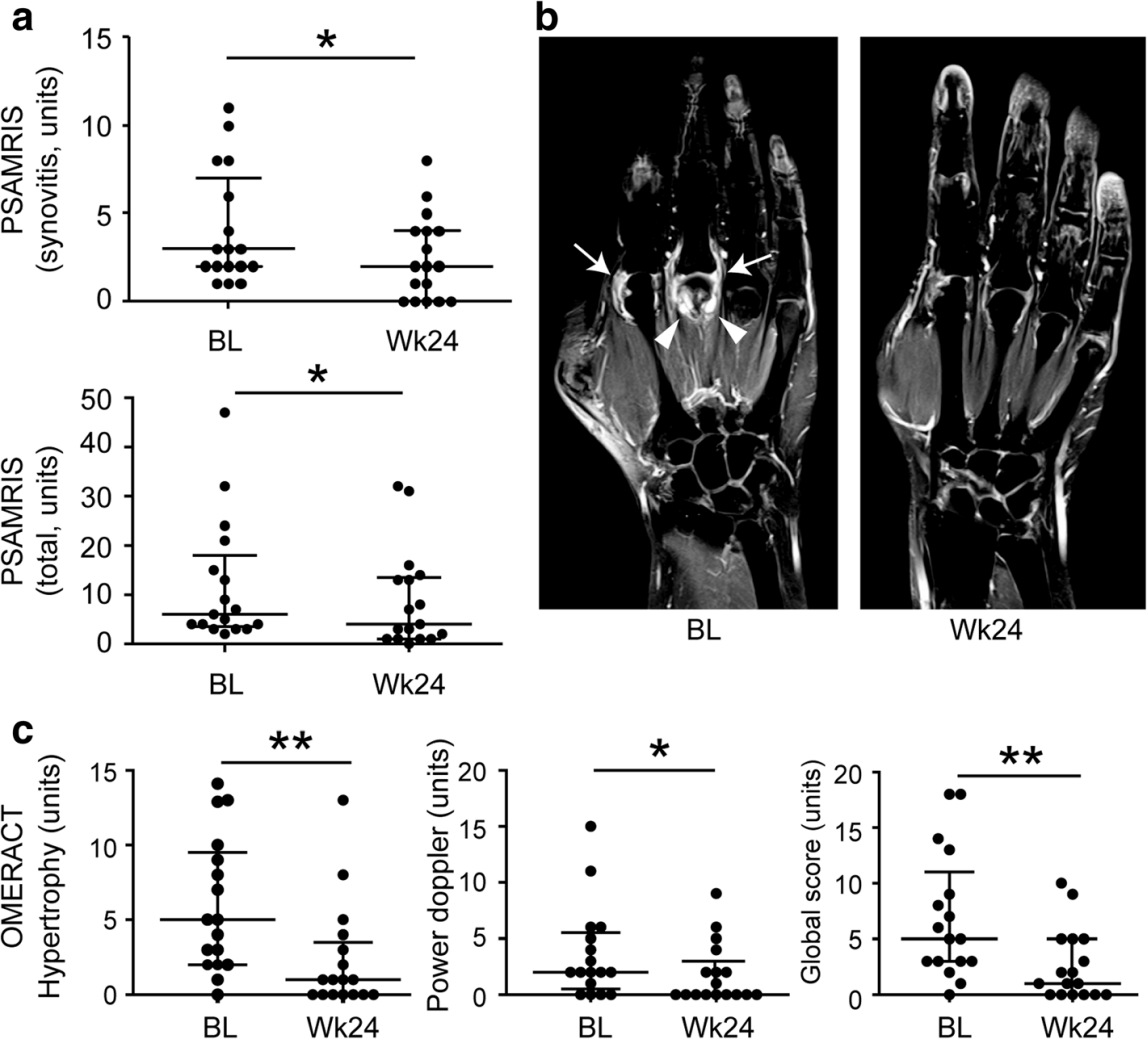
Effects of secukinumab treatment on the inflammatory changes in the joints of patients with psoriatic arthritis. **a** Comparison of inflammatory changes in the joints of psoriatic arthritis (PsA) patients using magnetic resonance imaging (MRI) at baseline (BL) and after 24 weeks (Wk24) of secukinumab treatment. Psoriatic arthritis MRI scores (PsAMRIS) for synovitis and total PsAMRIS scores are shown. **b** Representative coronal T1-weighted fat-suppressed post-gadolinium MRI images of the hand of the same patient with PsA at baseline and after 24 weeks are depicted. Baseline image shows synovitis (arrows) and periarticular inflammation at enthesial sites (arrowheads), which resolved after 24 weeks of secukinumab treatment. **c** Comparison of inflammatory changes in the joints of PsA patients using power Doppler ultrasound (PDUS) at baseline (BL) and after 24 weeks (Wk24) of secukinumab treatment. OMERACT ultrasound scores for synovial hypertrophy and power Doppler activity as well as global OMERACT ultrasound scores are shown. Data are presented as median and interquartile ranges. *P ≤0.05; **P ≤0.01

Sequential assessment of joint inflammation by MRI showed a significant decrease in global PsAMRIS (*P* = 0.039) and PsAMRIS synovitis score (*P* = 0.034) (Fig. 2a and b; Table 2; Additional file 1: Figure S1 and 2A; Additional file 1: Table S5) after 24 weeks of secukinumab treatment. Furthermore, periarticular inflammation completely disappeared (Table 2). With respect to PDUS assessment, global OMERACT EULAR ultrasound score (*P* = 0.005), synovial hypertrophy (*P* = 0.009), and power Doppler activity (*P* = 0.030) significantly improved after 24 weeks of secukinumab treatment (Table 2, Fig. 2c, Additional file 1: Figure S2B), confirming the data obtained by MRI. The effects of secukinumab on the reduction of synovitis in MRI and PDUS were not different between patients naïve or experienced to TNF inhibitors (Additional file 1: Table S2). When comparing previous or concomitant cDMARD treatment also no difference in response was detected (Additional file 1: Table S3 and S4).

**Table 2:** Changes of the inflammatory and structural parameters between baseline and week 24

Characteristic	Baseline	Week 24	*P* value
MRI			
PSAMRIS Synovitis (median,IQR)	3 (2, 7)	2 (0, 4)	0.034*
PSAMRIS Osteitis (median,IQR), (mean ± SD)†	0 (0, 5)†, 1.2 ± 3.1	0 (0, 0)†, 0.5 ± 1.3	0.180
PSAMRIS Periarticular (median,IQR) (mean ± SD)†	0 (0, 1)†, 0.7 ± 1.6	0 (0, 0)†, 0 ± 0	0.059
PSAMRIS Tenossynovitis (median,IQR) (mean ± SD)†	0 (0, 1.5)†, 1.2 ± 2.2	0 (0, 0)†, 0.7 ± 1.7	0.400
PSAMRIS Erosions (median, IQR)	1 (0, 3)	1 (0, 4)	0.167
PSAMRIS Proliferation (median,IQR) (mean ± SD)†	0 (0, 1)†, 1.2 ± 2.6	0 (0, 1)†, 1.2 ± 2.6	0.655
Total PSAMRIS (median, IQR)	6 (3.5, 18)	4 (1, 13.5)	0.039*
PDUS			
OMERACT Hypertrophy (median, IQR)	5 (2, 9.5)	1 (0, 3.5)	0.009**
OMERACT Effusion (median, IQR) (mean ± SD)†	4 (0.5, 6.5)†, 4.1 ± 3.9	0 (0, 4)†, 2.3 ± 3.2	0.084
OMERACT Power Doppler (median, IQR) (mean ± SD)†	2 (0.5, 5.5)†, 3.6 ± 4.1	0 (0, 3)†, 1.8 ± 2.7	0.030**
Global OMERACT (median, IQR)	5 (3, 11)	1 (0, 5)	0.003**
HR-pQCT			
Erosion Number (median, IQR)	2 (0.5, 4.5)	2 (1, 4)	0.059
Erosion Volume (median, IQR)	3.29 (0.40, 14.68)	3.87 (0.41, 16.7)	0.859
Proliferation Grade (median, IQR)	2 (1,2)	2 (1.5, 2)	0.083

*HR-pQCT* high-resolution peripheral quantitative computed tomography, *IQR* interquartile range, *MRI* magnetic resonance imaging, *OMERACT* outcome measures in rheumatoid arthritis clinical trials, *PDUS* power Doppler ultrasound, *PSAMRIS* psoriatic arthritis magnetic resonance imaging scoring system. †Additionally reported as mean and standard deviation (mean ± standard deviation) because median was equal to zero. Data are based on 17 psoriatic arthritis patients with complete baseline and 24 week data. Wilcoxon signed-rank test. *P ≤0.05, **P ≤0.01

### Effects of secukinumab on bone erosions

**Fig. 3:**
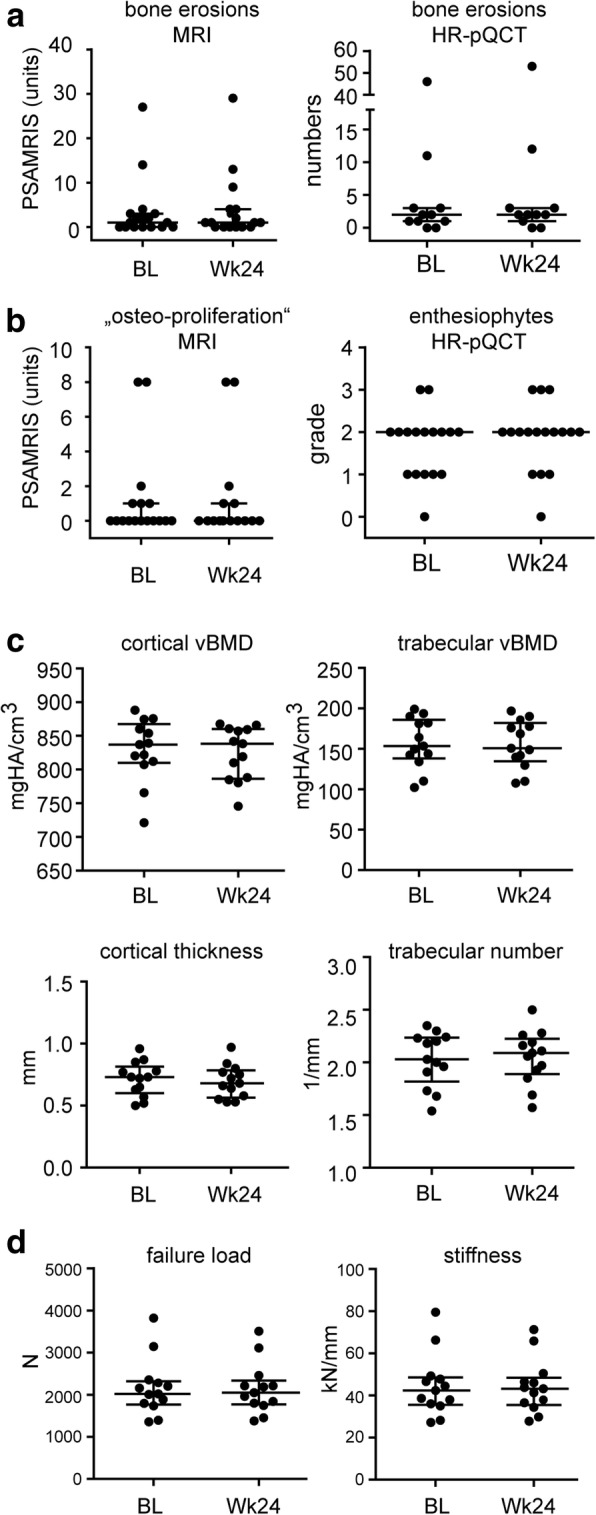
Effects of secukinumab treatment on the articular and extra-articular structural bone changes in patients with psoriatic arthritis. **a** Comparison of bone erosions in the joints of psoriatic arthritis (PsA) patients using magnetic resonance imaging (MRI, left) and high-resolution peripheral quantitative computed tomography (HR-pQCT, right) at baseline (BL) and after 24 weeks (Wk24) of secukinumab treatment. MRI data represent Psoriatic arthritis MRI scores (PsAMRIS) for erosions, and HR-pQCT data represent erosion volumes. **b** Comparison of enthesiophytes in the joints of PsA patients using MRI (left) and HR-pQCT (right) at baseline (BL) and after 24 weeks (Wk24) of secukinumab treatment. MRI data represent Psoriatic arthritis MRI scores (PsAMRIS) for proliferations, and HR-pQCT data represent enthesiophyte grades according to size. **c** Comparison of bone micro-structural data of the distal radius of PsA patients using HR-pQCT at baseline (BL) and after 24 weeks (Wk24) of secukinumab treatment. Cortical and trabecular volumetric bone density (vBMD), cortical thickness, and trabecular numbers are shown. **d** Comparison of biomechanical properties of radial bones of PsA patients using HR-pQCT measurements at baseline (BL) and after 24 weeks (Wk24) of secukinumab treatment. Data show failure load and bone stiffness based on micro-finite element analysis. Data are presented as median and interquartile ranges. Abbreviations: *kN* kiloNewton, *mgHA/cm*^*3*^ milligram of hydroxyapatite per cube centimeter, *N* Newton

We next examined whether secukinumab therapy arrests the progression of bone erosions in patients with PsA. Bone erosions were assessed at baseline and after 24 weeks of secukinumab treatment by using MRI and HR-pQCT. In MRI, PSAMRIS bone erosion score remained stable over the 24 weeks of treatment with no signs of progression (Table 2; Fig. 3a). In HR-pQCT, erosion numbers and erosion volume did not show any significant progression either (Table 2; Fig. 3a).

### Effects of secukinumab on enthesiophyte progression

Since enthesiophytes are a hallmark of structural damage in PsA, we were interested in whether the progression of these lesions is inhibited by secukinumab. In MRI, where osteoproliferation is also scored, no progression was found (Table 2; Fig. 3b). In HR-pQCT, which is more sensitive for enthesiophytes, no progression was detected (Table 2, Fig. 3b), suggesting that secukinumab also leads to an arrest of progression of anabolic bone changes in PsA.

### Effects of secukinumab on bone structure and functional properties

We next investigated bone structure and bone functional properties at the distal radius. Cortical and trabecular bone structure remained stable over 24 weeks of secukinumab treatment (Fig. 3c). Furthermore, cortical thickness, as the key microstructural parameter of cortical bone, and trabecular number, as the key microstructural parameter of trabecular bone, did not change between baseline and week 24 (Fig. 3c). Finally, functional bone parameters such as failure load and stiffness resembling bone strength according to micro-finite element analysis remained stable (Fig. 3d).

### Effects of secukinumab on clinical outcomes

DAS28-ESR declined from a median of 4.93 units at baseline to 2.93 units (*P* = 0.001) after 24 weeks, resembling low disease activity (Table 3). DAPSA declined from a median of 27.5 units at baseline to 5.7 units (*P* <0.001) after 24 weeks, also resembling low disease activity. Effects of DAPSA responses were similar between patients naïve or experienced to TNF inhibitors, while DAS28-ESR responses were better (*P* = 0.01; Mann–Whitney *U* test) in TNF inhibitor–naïve (2.4; 2.1–3.5) than experienced (1.5; 1.2–2.1) patients.

**Table 3:** Changes in signs and symptoms of psoriatic arthritis between baseline and week 24

Characteristic	Baseline	Week 24	*P* value
TJC 78 (median, IQR)	10 (6, 16.5)	1 (0, 3.5)	< 0.001**
SJC 76 (median, IQR), (mean ± SD)	4 (3, 7), 5.1 ± 2.5	0 (0, 0)†, 0.2 ± 0.4	< 0.001**
DAS28-ESR (median, IQR)	4.93 (4.08, 5.74)	2.93 (2.01, 3.70)	0.001**
DAPSA (median, IQR)	27.55 (22.53, 38.35)	5.73 (3.63, 3.70)	< 0.001**
PASI (median, IQR)	0.4 (0.2, 2.3)	0.1 (0, 1.3)	0.062
BSA% (median, IQR)	0.3 (0.2, 1.5)	0.2 (0, 1.9)	0.325
ACR20	-	82.4%	-
ACR50	-	52.9%	-
ACR70	-	35.3%	-
EULAR moderate	-	68.8%	-
EULAR good	-	25.0%	-
PSARC	-	88.2%	-
DAPSA minor#	-	11.8%	-
DAPSA moderate#	-	29.4%	-
DAPSA good#	-	35.3%	-
DAPSA high##	-	0%	-
DAPSA moderate##	-	17.6%	-
DAPSA low##	-	52.9%	-
DAPSA remission##	-	29.4%	-
MDA	-	46.2%	-

*ACR* American College of Rheumatology, *BSA%* percent body surface area, *DAPSA* disease activity in psoriatic arthritis score, *DAS28-ESR* disease activity score 28 based on erythrocyte sedimentation rate, *EULAR* European League Against Rheumatism, *IQR* interquartile range, *MDA* minimal disease activity, *PASI* psoriasis area and severity index, *PsARC* psoriatic arthritis response criteria, *SJC* swollen joint count, *TJC* tender joint count. #Minor (change ≥50%), moderate (≥75%), good (≥85%). ##Remission (DAPSA ≤4), low (>4 and ≤14), moderate (>14 and ≤28), high (>28). †Additionally reported as mean and standard deviation (mean ± standar deviation) because median was equal to zero. Data are based on 17 psoriatic arthritis patients with complete baseline and 24 week data. Wilcoxon signed-rank test. *P ≤0.05, **P ≤0.01

ACR 20, 50, and 70 were achieved in 82.4%, 52.9%, and 35.3% of patients, respectively. MDA was achieved in 46.2% of the patients after 24 weeks (Table 3). Patients reaching MDA had significantly better response in PDUS parameters of inflammation (OMERACT synovium hypertrophy and GLOESS, *P* <0.05) than those not reaching MDA.

## Discussion

The data from the PSARTROS study show that inhibition of IL-17A by secukinumab improves the local inflammatory changes in the joints of patients with PsA and also arrests the progression of structural changes. These data add knowledge to the existing clinical trial data on the effects of IL-17A inhibition on the signs and symptoms of PsA. Importantly, both MRI and PDUS analysis of the joints showed a consistent improvement of synovitis in PsA patients and no sign of structural progression, even with high-quality imaging such as MRI and HR-pQCT.

Clinical studies to date have shown compelling evidence that secukinumab improves pain, swelling, function, and quality of life. These data have led to the approval of secukinumab for the treatment of PsA. The effects of IL-17A inhibition by secukinumab on the local inflammatory and structural pathologies in the joints of patients with PsA are less well defined. Analysis of the joints by conventional radiography showed that secukinumab was associated with significant retardation of radiographic progression after 24 weeks [9]. This finding essentially supports the function of secukinumab as DMARD in PsA. However, overall radiographic progression rate is low, raising the question of whether conventional radiography may miss substantial aspects of structural pathology in PsA. Limitations associated with conventional radiography are (i) its resolution, (ii) the fact that anabolic features of bone damage are not analyzed, and (iii) the fact that no information on the inflammatory pathologies in the joints of patients with PsA is obtained.

MRI und PDUS are the techniques of choice to analyze inflammatory changes in patients with PsA, especially since instruments such as PSAMRIS and OMERACT ultrasound scores for grading have been developed [22, 23]. Unfortunately, these instruments have not been consistently implemented in evaluating the therapeutic responses of cytokine inhibitors in PsA. Herein, we show that it is feasible to find significant therapeutic responses by MRI und PDUS in even small numbers of patients with PsA. Hence, the burden of synovitis was consistently reduced by secukinumab irrespectively of whether MRI or PDUS was used to quantify inflammation. Furthermore, periarticular inflammation in the MRI, which, in part, resembles enthesitis, completely resolved upon secukinumab treatment. These data suggest that IL-17A inhibition allows full or at least partial resolution of inflammatory lesions in the joints. Given that clinical and imaging signs of inflammation are often not well aligned [30], these findings are particularly valuable, indicating that it is possible to clear inflammation from PsA joints by neutralizing IL-17A.

With respect to bone erosions, the observation that even highly sensitive techniques, such as MRI and particularly HR-pQCT, did not show any signs of progression provides solid evidence that the aforementioned reduction of synovitis by secukinumab is followed by protection from progression of structural damage. Direct effects of IL-17A inhibition on osteoclast differentiation may add to the observed protection from erosion in PsA. Even more importantly, PSARTROS provides the first evidence that enthesiophyte formation can be arrested in PsA. Hence, HR-pQCT assessment of this central structural change in patients with PsA [31] showed no signs of progression of bony spurs. In contrast, previous longitudinal data on enthesiophytes suggested progression of these anabolic bone lesions in both methotrexate- and TNF inhibitor-treated PsA patients [32]. Hence, these data provide the first evidence that enthesiophyte growth can be stopped in patients with PsA. These findings also support the increasingly recognized role of IL-17 in enthesitis [10].

IL-17 is a cytokine which negatively influences bone homeostasis by upregulating bone resorption [11–14] and inhibiting bone formation [19, 33]. Patients with PsA are characterized by bone loss and increased fracture risk [20]. We therefore took the opportunity to assess the effects of secukinumab on bone structure and strength. In this detailed analysis of bone mass, microstructure, and strength, no significant decline of bone parameters could be observed over a period of 24 weeks. These data represent the first longitudinal data on bone microstructure and strength during the treatment of arthritis and provide strong evidence that IL-17A targeting maintains the structural and functional properties of bone in patients with PsA.

Although this study provides the first comprehensive and in-depth imaging analysis of the joints of PsA patients during bDMARD therapy, several limitations have to be discussed. First, there is no placebo control arm, which does not completely rule out the possibility that in placebo-treated patients no structural progression would have occurred. However, inclusion of a placebo arm over 24 weeks would have been ethically challenging given that active PsA patients failing on methotrexate or biologic therapy were included. Furthermore, it seems unlikely that placebo treatment would have led to significant responses on synovitis. Second, this is an exploratory study with a small sample size using high-end imaging. Hence, generalizability of the data needs to be regarded with caution, and it cannot be excluded that, in larger samples, some patients progress despite secukinumab treatment. Nonetheless, in this study, significant effects on the inflammatory changes by MRI and PDUS were detected. Finally, long-term effects of secukinumab treatment on bone structure are not assessed by this study, which lasted for 24 weeks. Hence, especially the effects on bone structure may take more time than only 24 weeks and could have been missed.

## Conclusions

In summary, the PSARTROS study shows that targeting IL-17 by secukinumab effectively controls synovitis and leads to an arrest of catabolic as well as anabolic structural bone changes in patients with PsA. These data underline a causative role of IL-17 in triggering joint disease in the context of psoriasis.

## Additional files

Additional file 1: **Figure S1.** Examples of baseline and follow-up MRI and HR-pQCT images. **Figure S2.** Single patient analysis of MRI and PDUS changes. **Table S1.** Pattern of joint involvement at baseline. **Table S2.** Imaging data in PsA patients with and without pre-exposure to TNF inhibitors. **Table S3.** Imaging data in PsA patients with respect to concomitant and previous csDMARD treatments. **Table S4.** Imaging data in PsA patients with respect to concomitant methotrexate treatment. **Table S5.** Proportion of patients with improvement in MRI, PDUS, and clinical outcome. (DOCX 3094 kb)

## Abbreviations

ACR: American Colleague of Rheumatology
bDMARD: Biological disease-modifying antirheumatic drug
BSA: Body surface area
DAPSA: Disease Activity in psoriatic arthritis
DAS: Disease Activity Score
DMARD: Disease-modifying antirheumatic drug
ESR: Erythrocyte sedimentation rate
EULAR: European League Against Rheumatism
GLOESS: Global OMERACT EULAR Ultrasound Synovitis Score
HR-pQCT: High-resolution peripheral quantitative computer tomography
IL-17: Interleukin-17
IQR: Interquartile range
MCP: Metacarpophalangeal
MDA: Minimal disease activity
MRI: Magnetic resonance imaging
OMERACT: Outcome Measures in Rheumatoid Arthritis Clinical Trials
PASI: Psoriasis area severity index
PDUS: Power Doppler ultrasound
PIP: Proximal interphalangeal
PsA: Psoriatic arthritis
SJC: Swollen joint count
TJC: Tender joint count
TNF: Tumor necrosis factor
