# Supplementary material for: Resolution of synovitis and arrest of catabolic and anabolic bone changes in patients with psoriatic arthritis by IL-17A blockade with secukinumab: results from the prospective PSARTROS study
Source: Arthritis Res Ther. 2018 Jul 27;20:153. doi: 10.1186/s13075-018-1653-5 (PMC6063019; doi:10.1186/s13075-018-1653-5)
Supplement: Supplementary file 1 — Figure S1. Examples of baseline and follow-up MRI and HR-pQCT images. Figure S2. Single patient analysis of MRI and PDUS changes. Table S1. Pattern of joint involvement at baseline. Table S2. Imaging data in PsA patients with and without pre-exposure to TNF inhibitors. Table S3. Imaging data in PsA patients with respect to concomitant and previous csDMARD treatments. Table S4. Imaging data in PsA patients with respect to concomitant methotrexate treatment. Table S5. Proportion of patients with improvement in MRI, PDUS, and clinical outcome. (DOCX 3094 kb) [file 13075_2018_1653_MOESM1_ESM.docx]

**Supplementary Figure and Tables**

**Supplementary Figures**

**Figure S1: Examples of baseline and follow-up MRI and HR-pQCT images**


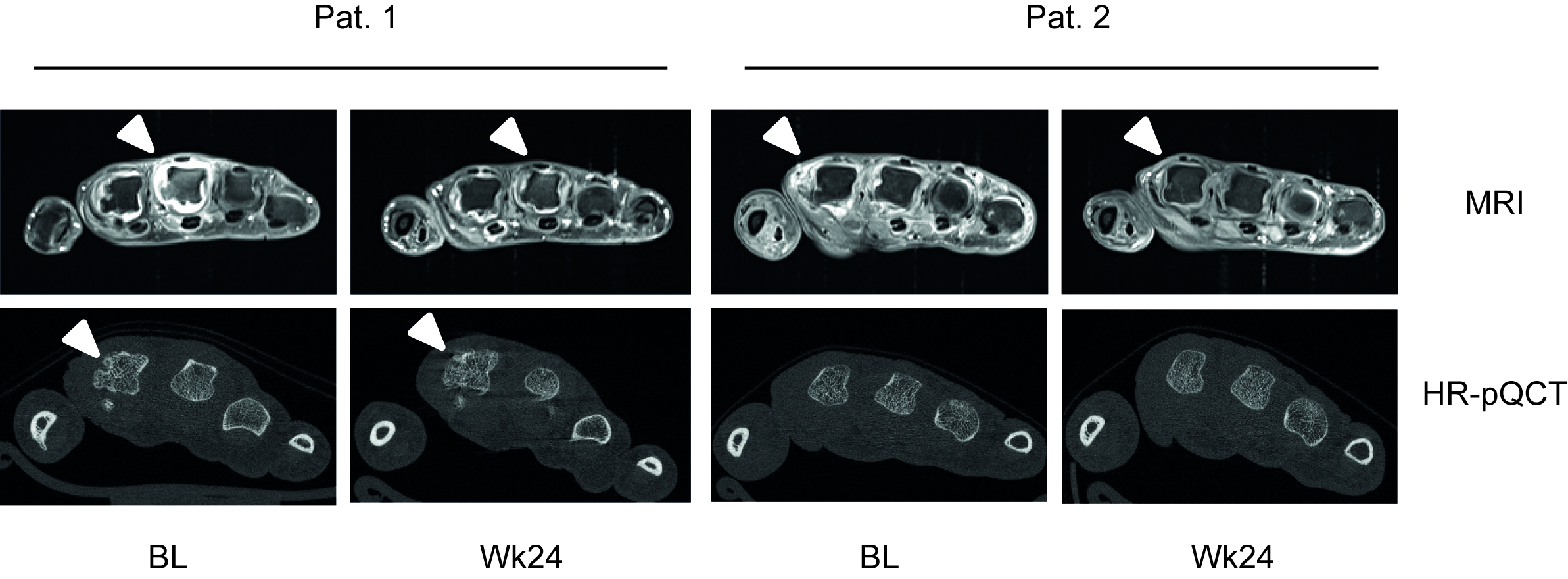


Upper row shows T1weighted fat-suppressed post-gadolinium sagittal MRI images of two patients. First and third columns show baseline (BL) images, while second and forth columns show data from week 24 (Wk24). Arrowheads in the top row show inflammation, which resolves after 24 weeks of secukinumab treatment. Lower row shows high-resolution peripheral quantitative computed tomography (HR-pQCT) images. First patient (left) shows an erosion in MCP2 head which remains stable after treatment (arrowhead). Patient 2 (right) shows no erosions.

**Figure S2: Single patient analysis of MRI and PDUS changes**


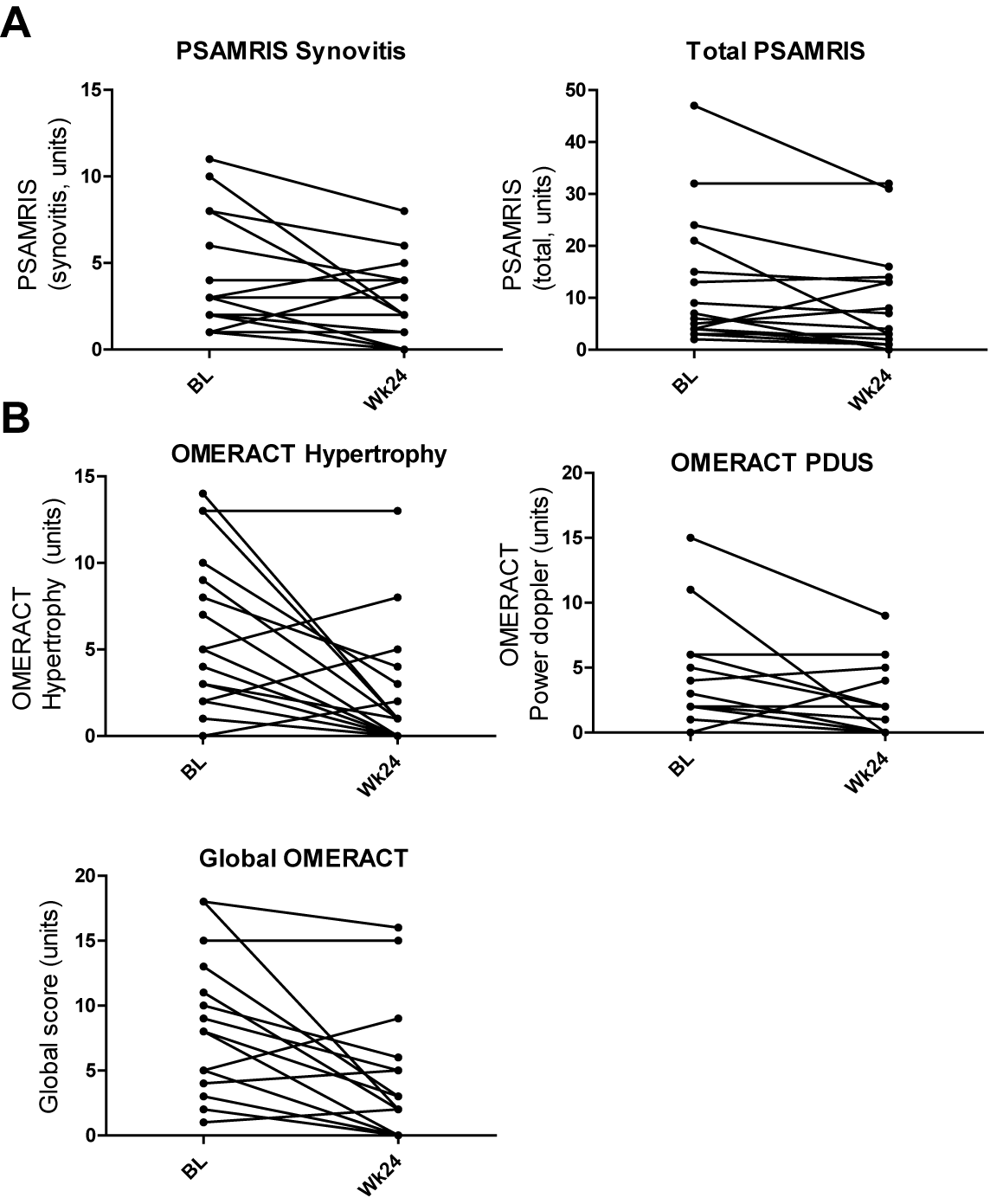


1. Psoriatic arthritis MRI scores (PsAMRIS) for synovitis (left graph) and total PsAMRIS scores (right graph) at baseline (BL) and after 24 weeks of secukinumab treatment. (B) OMERACT ultrasound scores for synovial hypertrophy (left graph) and Power Doppler activity (right graph) as well as global OMERACT ultrasound scores (bottom graph) at baseline (BL) and after 24 weeks of secukinumab treatment. Each line represents one patient. Data are based on 17 psoriatic arthritis patients with complete baseline and 24 week data.

**Table S1: Pattern of joint involvement at baseline**

| **Arthritis Type** |  |
| --- | --- |
| Oligoarthritis (≤4 joints) (% of patients) | 55% |
| Polyarthritis (>4 joints) (% of patients) | 45% |
|  |  |
| **Clinical Hand Involvement** |  |
| Joint tenderness (% of patients) | 100% |
| Tender Joint Count (Median, IQR) | 5 (3, 9) |
| Joint swelling (% of patients) | 95% |
| Swollen Joint Count (Median, IQR) | 3 (1, 4.5) |
|  |  |
| **Hand Involvement in imaging** |  |
| MRI (% of patients) | 100% |
| PDUS (% of patients) | 88,2% |

MRI, magnetic resonance imaging, PDUS, Power Doppler ultrasound.

**Table S2: Imaging data in PsA patients with and without pre-exposure to TNF inhibitors**

| **Characteristic** | **TNF naive** | **TNF exposed** | **P Value** |
| --- | --- | --- | --- |
| **MRI** |  |  |  |
| Δ PSAMRIS Synovitis (median, IQR) | 1 (-1, 4.5) | 2 (0.5, 2) | 0.541 |
| Δ PSAMRIS Osteitis (median, IQR) | 0 (0, 0) | 0 (0, 1.5) | 1.000 |
| Δ PSAMRIS Periarticular diff (median, IQR) | 0 (0, 1) | 0 (0, 1.5) | 0.481 |
| Δ PSAMRIS Tenosynovitis (median, IQR) | 0 (0, 0.5) | 0 (0, 1.5) | 0.963 |
| Δ Total PSAMRIS (median, IQR) | 2 (-1.5, 5) | 2 (0.5, 6) | 0.541 |
| **Ultrasound** |  |  |  |
| Δ OMERACT Hypertrophy (median, IQR) | 2 (-0.5, 6) | 3.5 (0.5, 11) | 0.481 |
| Δ OMERACT Effusion (median, IQR) | 1 (-0.5, 2) | 1.5 (0, 8) | 0.167 |
| Δ OMERACT Power Doppler (median, IQR) | 2 (-0.5, 6) | 3.5 (0.5, 11) | 0.481 |
| Δ GLOESS (median, IQR) | 3 (0.5, 5) | 4.5 (0.75, 9.75) | 0.370 |

OMERACT, outcome measures in rheumatoid arthritis clinical trials; PSAMRIS, psoriatic arthritis magnetic resonance imaging scoring system; MRI, magnetic resonance imaging, PDUS, Power Doppler ultrasound; IQR: interquartile range; Data are based on 17 psoriatic arthritis patients with complete baseline and 24 week data.

**Supplementary Table 3: Imaging data in PsA patients with respect to concomitant and previous csDMARD treatments**

|  | **no concomitant csDMARDs** | **concomitant csDMARDs** | **P Value** |
| --- | --- | --- | --- |
| **MRI** |  |  |  |
| Δ PSAMRIS Synovitis (median, IQR) | 1 (-1, 3) | 2 (0.5, 2) | 0.423 |
| Δ PSAMRIS Osteitis (median, IQR) (mean±SD) † | 0 (0, 0) (1.11 ± 3.33) | 0 (0, 0) (0.25 ± 0.71) | 0.423 |
| Δ PSAMRIS Periarticular (median, IQR) (mean±SD) † | 0 (0, 2) | 0 (0, 0) (0.25 ± 0.71) | 1.000 |
| Δ PSAMRIS Tenosynovitis (median, IQR) | 0 (0, 0.5) | 0 (0, 1.5) | 0.963 |
| Δ Total PSAMRIS (median, IQR) | 2 (-1.5, 9) | 2 (0.5, 6) | 0.606 |
| **Ultrasound** |  |  |  |
| Δ OMERACT Hypertrophy (median, IQR) | 4 (-0.5, 7) | 2.5 (0.5, 7) | 0.963 |
| Δ OMERACT Effusion (median, IQR) | 0 (-0.5, 1.5) | 2.5 (0, 8) | 0.423 |
| Δ OMERACT Power Doppler (median, IQR) | 4 (-0.5, 7) | 2.5 (0.5, 7) | 0.963 |
| Δ GLOESS diff (median, IQR) | 4 (0.5, 6.5) | 3.5 (0.5, 8) | 0.963 |
|  |  |  |  |
|  | **no previous csDMARDs** | **previous csDMARDs** | **P Value** |
| **MRI** |  |  |  |
| Δ PSAMRIS Synovitis (median, IQR) | 1 (0, 2) | 2 (0, 3.75) | 0.417 |
| Δ PSAMRIS Osteitis (median, IQR) (mean ±SD) † | 0 (0, 0) (0 ± 0) | 0 (0, 0.5) | 0.536 |
| Δ PSAMRIS Periarticular (median, IQR) (mean ±SD) † | 0 (0, 0) (0 ± 0) | 0 (0, 2) | 0.193 |
| Δ PSAMRIS Tenosynovitis (median, IQR) (mean ±SD) † | 0 (0, 0) (1 ± 0) | 0 (-0.5, 0.25) | 0.475 |
| Δ Total PSAMRIS (median, IQR) | 1 (-1, 2) | 2 (1.5, 10) | 0.133 |
| **Ultrasound** |  |  |  |
| Δ OMERACT Hypertrophy (median, IQR) | 4 (-3, 7) | 2 (0.75, 8.25) | 0.887 |
| Δ OMERACT Effusion (median, IQR) | 0 (-1, 5) | 1.5 (0, 3.25) | 0.601 |
| Δ OMERACT Power Doppler (median, IQR) | 4 (-3, 7) | 2 (0.75, 8.25) | 0.887 |
| Δ GLOESS diff (median, IQR) | 4 (-1, 8) | 4 (1.5, 6.25) | 0.740 |

OMERACT, outcome measures in rheumatoid arthritis clinical trials; PSAMRIS, psoriatic arthritis magnetic resonance imaging scoring system; MRI, magnetic resonance imaging, PDUS, Power Doppler ultrasound; IQR: interquartile range; †additionally reported as mean and standard deviation (mean± SD) because median was equal to zero. Data are based on 17 psoriatic arthritis patients with complete baseline and 24 week data. csDMARD, conventional synthetic disease modifying anti-rheumatic drugs.

**Supplementary Table 4: Imaging data in PsA patients with respect to concomitant methotrexate treatment**

| **Characteristic** | **no MTX use** | **MTX use** | **P Value** |
| --- | --- | --- | --- |
| **MRI** |  |  |  |
| Δ PSAMRIS Synovitis (median, IQR) | 1.5 (-2, 2.75) | 2 (0, 2.5) | 0.871 |
| Δ PSAMRIS Osteitis (median, IQR) (mean ±SD) † | 0 (0, 1.5) | 0 (0, 0) (0.77 ± 2.77) | 0.703 |
| Δ PSAMRIS Periarticular (median, IQR) | 0 (0, 1.5) | 0 (0, 1) | 1.000 |
| Δ PSAMRIS Tenosynovitis (median, IQR) | 0 (-1.5, 0) | 0 (0, 1.5) | 0.296 |
| Δ Total PSAMRIS (median, IQR) | 1.5 (-6.5, 2) | 2 (0, 7.5) | 0.296 |
| **Ultrasound** |  |  |  |
| Δ OMERACT Hypertrophy (median, IQR) | 5.5 (-1.25, 11.5) | 2 (0.5, 6) | 0.477 |
| Δ OMERACT Effusion (median, IQR) | 0.5 (-0.75, 8.5) | 1 (0, 3.5) | 0.956 |
| Δ OMERACT Power Doppler (median, IQR) (mean ±SD) Δ † | 5.5 (-1.25, 11.5) | 2 (0.5, 6) | 0.477 |
| Δ GLOESS (median, IQR) | 6 (0.25, 14) | 3 (1, 5) | 0.477 |

OMERACT, outcome measures in rheumatoid arthritis clinical trials; PSAMRIS, psoriatic arthritis magnetic resonance imaging scoring system; MRI, magnetic resonance imaging, PDUS, Power Doppler ultrasound; IQR: interquartile range; †additionally reported as mean and standard deviation (mean± SD) because median was equal to zero. Data are based on 17 psoriatic arthritis patients with complete baseline and 24 week data. MTX, methotrexate.

**Supplementary Table 5: Proportion of patients with improvement in MRI, PDUS and clinical outcome**

| **Characteristic** | **Improvement** | **Worsening** | **Stable** |
| --- | --- | --- | --- |
| **MRI** |  |  |  |
| PSAMRIS Synovitis | 64.7% | 11.8% | 23.5% |
| Total PSAMRIS | 70.6% | 17.6% | 11.8% |
| **PDUS** |  |  |  |
| OMERACT Hypertrophy | 76.5% | 17.6% | 5.9% |
| OMERACT Power Doppler | 76.5% | 17.6% | 5.9% |
| GLOESS | 76.5% | 17.6% | 5.9% |
| **Clinical** |  |  |  |
| DAS28-ESR | 93.8% | 6.2% | 0% |
| DAPSA | 94.1% | 5.9% | 0% |

OMERACT, outcome measures in rheumatoid arthritis clinical trials; PSAMRIS, psoriatic arthritis magnetic resonance imaging scoring system; MRI, magnetic resonance imaging, PDUS, Power Doppler ultrasound. DAS28-ESR, disease activity score 28 based on erythrocyte sedimentation rate; DAPSA, disease activity in psoriatic arthritis score. Improvement was defined as a lower score in comparison to the baseline measurement. Vice versa worsening was defined as a larger score compared to baseline. Data are based on 17 psoriatic arthritis patients with complete baseline and 24 week data.
